# RETRACTED: Atta et al. Synthesis of New Magnetic Crosslinked Poly (Ionic Liquid) Nanocomposites for Fast Congo Red Removal from Industrial Wastewater. *Nanomaterials* 2019, *9*, 1286

**DOI:** 10.3390/nano15241881

**Published:** 2025-12-15

**Authors:** Ayman M. Atta, Abdelrahman O. Ezzat, Yaser M. Moustafa, Nourah I. Sabeela, Ahmed M. Tawfeek, Hamad A. Al-Lohedan, Ahmed I. Hashem

**Affiliations:** 1 Chemistry Department, College of Science, King Saud University, Riyadh 11451, Saudi Arabia; ao_ezzat@yahoo.com (A.O.E.); noonah-37@hotmail.com (N.I.S.); atawfik@ksu.edu.sa (A.M.T.); hlohedan@ksu.edu.sa (H.A.A.-L.); 2 Egyptian Petroleum Research Institute, Nasr City 1772, Cairo, Egypt; ymoustafa12@yahoo.com; 3 Chemistry Department, Faculty of Science, Ain Shams University, Abasia 11566, Cairo, Egypt; emyhashem2004@yahoo.com

The journal retracts the article “Synthesis of New Magnetic Crosslinked Poly (Ionic Liquid) Nanocomposites for Fast Congo Red Removal from Industrial Wastewater” [1] cited above.

Following publication, concerns were brought to the attention of the Editorial Office regarding the integrity of an image presented in this publication [1].

Adhering to our standard procedure, the Editorial Office and Editorial Board conducted an investigation that confirmed image modification and duplication between Figure 4a published in [1] and Figure 2b in [2]; Figure 5a published in [1] and Figure 6c in [3]; and Figure 5b published in [1] and Figure 2b in [4], presenting different experimental conditions. While the authors collaborated with this process, raw material meeting the journal’s requirements for original images (https://www.mdpi.com/journal/nanomaterials/instructions#oriimages) could not be provided for Editorial Board Evaluation. Consequently, the Editorial Board has lost confidence in the reliability of the findings and has decided to retract this publication, as per MDPI’s retraction policy (https://www.mdpi.com/ethics#_bookmark30).

This retraction was approved by the Editor-in-Chief of the journal *Nanomaterials*.

The authors did not agree to this retraction.
